# Geospatial and demographic patterns of SARS-CoV-2 spread in Massachusetts from over 130,000 genomes

**DOI:** 10.1101/2025.04.04.25324273

**Published:** 2025-04-06

**Authors:** Gage K. Moreno, Taylor Brock-Fisher, Lydia A. Krasilnikova, Steve F. Schaffner, Meagan Burns, Carolyn E. Casiello, Katelyn S. Messer, Brittany Petros, Ivan Specht, Katherine C. DeRuff, Katherine J. Siddle, Christine Loreth, Nicholas A. Fitzgerald, Heather M. Rooke, Stacey B. Gabriel, Sandra Smole, Shirlee Wohl, Daniel J. Park, Lawrence C. Madoff, Catherine M. Brown, Bronwyn L. MacInnis, Pardis C. Sabeti

**Affiliations:** 1 Broad Institute of Harvard and MIT, Cambridge, MA, USA; 2 Department of Organismic and Evolutionary Biology, Harvard University, Cambridge, MA, USA; 3 Howard Hughes Medical Institute, Chevy Chase, MD, USA; 4 Department of Immunology and Infectious Diseases, Harvard T.H. Chan School of Public Health, Harvard University, Boston, MA, USA; 5 Massachusetts Department of Public Health, Boston, MA, USA; 6 Harvard-MIT Program in Health Sciences and Technology, Cambridge, MA, USA; 7 Harvard/MIT MD-PhD Program, Boston, MA, USA; 8 Department of Molecular Microbiology and Immunology, Brown University, Providence, RI, USA; 9 Brigham and Women’s Hospital, Boston, Massachusetts, USA; 10 Massachusetts Consortium for Pathogen Readiness, Boston, MA, USA

## Abstract

Despite intensive study, gaps remain in our understanding of SARS-CoV-2 transmission patterns during the COVID-19 pandemic, in part due to limited contextual metadata accompanying most large genomic surveillance datasets. We analyzed over 130,000 SARS-CoV-2 genomes, over 85,000 with matched epidemiological data, collected in Massachusetts from November 2021 to January 2023, to investigate viral transmission dynamics at high resolution. The data were drawn from diagnostic testing at >600 facilities representing schools, workplaces, public testing, and other sectors, and encompass the emergence of six major viral lineages, each representing a new outbreak. We found urban areas as key hubs for new lineage introduction and interurban transmission as facilitating spread throughout the state. Young adults, especially those on college campuses, served as early indicators of emerging lineage dominance. Resident-aged populations in college campuses and nursing homes exhibited a higher likelihood of being linked to within-facility transmission, while staff-aged at those facilities were more linked to their surrounding community. Individuals with recent vaccine doses, including boosters, had a lower likelihood of initiating transmission. This dataset shows the value of linking genomic and epidemiologic data at scale for higher resolution insights into viral dynamics and their implication for public health strategy.

## Introduction

Extensive genomic surveillance provided crucial information about the evolution and spread of SARS-CoV-2 during the COVID-19 pandemic. As the proximal goal of most large-scale genomic surveillance efforts was to identify and track emerging variants of concern, which requires minimal associated metadata, the association of more complete metadata to further explore genomic datasets was rare. The few existing studies of large SARS-CoV-2 genome datasets that have incorporated epidemiological data have yielded insights into infection risk^1^, viral evolution^2^, the effect of travel patterns on pandemic waves^3,4^, and transmission within and between age groups^5^. Here we explore a unique and rich dataset combining viral genomic and paired comprehensive epidemiological data to understand SARS-CoV-2 transmission across multiple variant waves.

The Broad Institute’s clinical laboratory provided COVID-19 diagnostic testing and SARS-CoV-2 sequencing throughout the acute phase of the pandemic, ultimately responsible for over 37 million SARS-CoV-2 tests and 175,000 genomes (~5% of tests and of genomes in the United States) by early 2023^6,7^, with a focus in the Broad’s home state of Massachusetts. In parallel, the Massachusetts Department of Public Health (MDPH) and municipal public health departments collected extensive epidemiological data on COVID-19 cases in the state. Here we present an analysis of over 130,000 SARS-CoV-2 genomes collected in Massachusetts between November 1, 2021, and January 17, 2023 (spanning the late Delta and the BA.1, BA.2, BA.4/5, BQ.1, and XBB Omicron waves), over 85,000 of which have accompanying epidemiological data including age, gender, vaccination status, city of residence, testing sector, and testing facility.

Using these data, we investigated open questions that can uniquely be addressed with such an extensive dataset of paired epidemiological and genomic data. By integrating geospatial data from six SARS-CoV-2 lineages, we examined how new lineages entered and disseminated within the state. Using facility-level genome collection data, we analyzed transmission patterns across different sectors and subpopulations. Additionally, we assessed the effect of vaccination and booster doses on infection and transmission. Our study provides a high resolution view of the complexity of SARS-CoV-2 transmission that may inform future public health surveillance and response strategies more broadly.

## Results

### Over 85,000 SARS-CoV-2 genomes paired to individual epidemiological data

As part of the US CDC’s National SARS-CoV-2 Strain Surveillance (NS3) program, the Broad Institute generated 134,785 high-quality (≥80% complete) SARS-CoV-2 genomes from residual positive diagnostic samples collected from Massachusetts residents between November 1, 2021, and January 17, 2023. These samples were obtained from the Broad Institute clinical laboratory’s large-scale, PCR-based COVID-19 testing program^8^, which served public testing venues as well as testing carried out in educational, medical, and congregate living settings.

**Figure 1.**
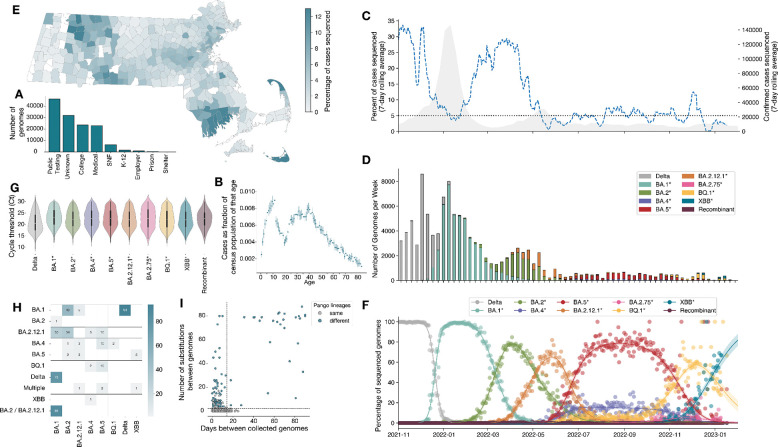
Trends in SARS-CoV-2 genomic surveillance in Massachusetts during study period. A) Histogram of the number of genomes collected from each testing sector. B) Cases in dataset from public testing by age, shown as the fraction of the Massachusetts census population of that age. C) Gray shaded region is the 7-day rolling average of SARS-CoV-2 case counts as reported to MDPH between November 1 2021, and January 17 2023. The blue dashed line represents the 7-day rolling average of the percent of cases sequenced as part of this study on a given day. The horizontal black line is at 5% of confirmed cases sequenced, which represents the US CDC’s national SARS-CoV-2 surveillance program target of sequencing ≥5% of confirmed cases. D) Number of sequenced genomes attributed to each lineage by week in the complete dataset. E) Percent of confirmed cases in each municipality that were sequenced. F) Multinomial regression showing logistic growth of Delta and of Omicron sublineages in the complete dataset. Shaded regions represent 95% confidence intervals. G) Violin plot of cycle threshold (Ct) values attributed to each lineage. H) A heatmap of the number of mixed lineage infections. Along the x-axis the lineage that was called in the consensus. Along the y-axis is the lineage detected at sub-consensus frequencies. I) Scatter plot of the number of days between genomes collected from the same individuals and the number of mutations separating those genomes. Points are colored by if the Pango lineage was the same (gray) or different (blue). The horizontal dashed line is at 2 mutations and the vertical dashed line is at 14 days. Individuals outside of this window may represent evolution in persistently infected individuals.

Genomes were sequenced from individual samples collected at 666 facilities statewide, which we have classified into 7 testing sectors, (Fig 1A): 29 public testing facilities (35% of genomes), 101 colleges (19%), 52 medical facilities (17%), 301 skilled nursing facilities (SNFs, 5%), 44 primary and secondary schools (1%), 23 workplaces (<1%), and 117 testing facilities of unknown sector (22%). We obtained deidentified paired epidemiological data—including age, sex, city of residence, and vaccination information (brand and date of each dose)—for 85,125 unique individuals in the dataset; unless otherwise indicated, this was the core dataset used for analysis. For 20,259 of individuals, symptoms and outcomes were also available (Table 1, Sup Fig 1).

Samples for sequencing were selected to be broadly representative of the contributing testing facilities (Fig 1B), and at a rate that generally corresponded to statewide case count trends, averaging 10.5% of all confirmed cases as reported to MDPH per week between November 2021 and January 2023 (Fig 1C, 1D). Some sectors were known to be enriched for specific subpopulations—children (schools), young adults (colleges), and older adults (SNFs). All subsequent analyses make the assumption that public testing venues, which were openly accessible regardless of the presence of symptoms, were representative of the state population as a whole. Of the 351 municipalities in the state, 87% (304) had genomes from ≥1% of all confirmed cases in the municipality (Fig 1E). The majority of genomes were derived from samples collected from residents of the state’s three most populous metropolitan areas: the capital city Boston (n=25,094, 28.6% of total dataset) and the regional hub cities Springfield (n=5,701, 6.5%) and Worcester (n=3,585, 4.1%). In total, the genomes comprised 492 named Pango lineages, which fell into 7 dominant lineages: Delta (B.1.1.617.2*) and the BA.1, BA.2, BA.4, BA.5, BQ.1, and XBB Omicron sub-variants (Fig 1F). As seen in previous studies^9,10^, clinical diagnostic PCR cycle threshold (Ct) values were significantly lower for Delta than for the Omicron sublineages, with little variation among the latter (Fig 1G, Sup Fig 2).

For a subset of the individuals with associated epidemiological data (2,714 of 85,125), genomes were obtained from multiple samples collected within 90 days of one another (Sup Fig 3A–3C), the timeframe over which tests per individual were tracked in the public health system. Of the 2,664 individuals with multiple samples collected specifically within 14 days, the CDC definition of being from the same infection^11^, 97% (n=2,590/2,664) retained the same Pango lineage across multiple samples in those 14 days, while 3% (n=85) were infected with different lineages. (11 individuals had more than 2 samples that fell into both categories.) Among the remaining 56 individuals with samples collected between 14 and 90 days apart, 24 were infected with the same Pango lineage; the high similarity between the paired genomes (a mean of 0.5 substitutions between genomes collected a mean of 21 days apart) suggests that most represent single persistent infections. The remaining 32 individuals had different Pango lineages, indicating a reinfection within 90 days (Fig 1I, Sup Fig 3C).

We also looked for instances of simultaneous infection with more than one lineage by identifying samples with two or more-clade defining mutations present only as minor alleles. In the 85,125 individual samples, we identified 434 (0.5%) occurrences of infections comprising more than one lineage (Fig 1H, Sup Fig 3D). Most of these were infections with Delta and BA.1*, likely reflective of the very high infection rate during the early BA.1 wave.

### High resolution temporal and spatial mapping of viral spread

**Figure 2.**
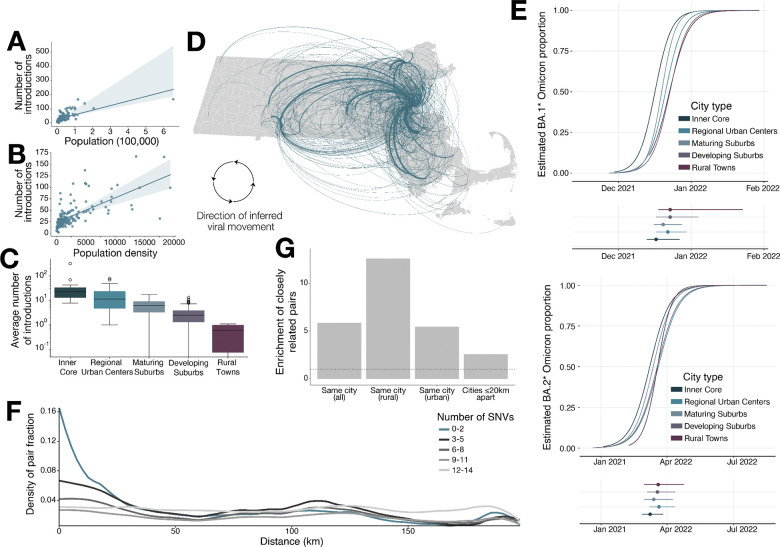
Viral movements within Massachusetts driven by more populous and dense populations. A) Scatterplot of population density of each municipality and the total number of introductions observed into that municipality. B) Scatterplot of population of each municipality and the total number of introductions observed into that municipality. C) Boxplot of the number of introductions (averaged across 10 trees, and all 6 lineages) into each municipality, binned by municipality type. D) Observed movements between each Massachusetts municipality. Line thickness corresponds to the number of movements observed from the phylogenetic tree. Directionality of movement is in a clockwise direction. E) Logistic regression plots of BA.1* (top) and BA.2* (bottom) with 95% confidence interval of 50% dates. F) Density plot of the proportion of pairs within a given genetic distance (as a fraction of all pairs) across geographic distance, closely related pairs (defined as 2 or fewer SNPs difference) shown in blue. G) Barplot of the relative enrichment of the proportion of closely related virus pairs among pairs from the same municipality, the same municipality among only municipalities classified as rural or developing suburbs, the same municipality among only municipalities classified as mature suburbs, regional urban centers, and the inner core, and municipalities within 20 km. The dashed line at 1 represents the background relatedness among each category.

Because our dataset covered the introduction of six distinct Omicron sub-variants with high geographic resolution, we were able to examine patterns in the introduction, growth, and spread of new lineages. We found that new lineages from out of state were introduced preferentially into more urban areas (Sup Fig 4), while the source of their movement within the state showed a similar urban bias. More precisely, the probability that a sampled virus represented a new introduction into the state was significantly correlated with both the population size and density of the municipality where it was collected. Similarly, for intrastate movement, this probability was correlated with the population density of the source municipality. See Methods for details. This bias, combined with the larger size of urban municipalities, meant that they played an outsized role as both the recipients of new external introductions (Fig 2A, 2B, 2C, Sup Fig 4) and the source of intrastate movement of new lineages (Fig 2D). Because urbanicity itself was evidently a factor in how lineages spread across the state, for subsequent analyses we classified municipalities by how urban they were, incorporating both population size and density; the 5 categories were the urban inner core of the Boston metropolitan area, regional urban centers, mature suburbs, developing suburbs, and rural towns^12^.

For the two lineages for which we had enough data, BA.1* and BA.2*, we looked at how quickly each lineage became established (reached 50% frequency) across municipality types. We found that both lineages were established first in the Inner Core, followed by the Maturing Suburbs and then the Regional Urban Centers. That is, new variants reached high frequency in the immediate suburbs of Boston faster than in other regional cities, even though external introductions were more common in the latter, suggesting the importance of local viral diffusion from the initial introduction into Boston in the early growth of newly introduced variants (Fig 2E).

We next investigated patterns and rates of viral spread as a function of distance. To do this, we looked at all pairs of viruses of the same lineage collected within 10 days (~2 serial intervals)^13^ of each other through public testing. (BA.1*, which had a rapid and nearly clonal rise, showed too little geographic structure to be informative about viral spread and is omitted from this analysis; Sup Fig 5.) For these pairs we examined how the genetic (Hamming) distances between the two genomes varied with the geographic distance between the infected individuals, the latter calculated from their municipalities of residence. The most striking feature was the rapid decline with distance in how likely two viruses were to be very similar to one another, reflecting the effect of local transmission (Fig 2F). Because this sharp dropoff is only present for the most genetically similar viruses — those differing by ≤2 substitutions — we defined viruses meeting that criterion as ‘closely related’, i.e. they lie close together in a transmission chain, for use in subsequent analyses, as seen in other studies^5,14,15^.

Also notable was that viruses were more likely to be related to each other if they both came from urban areas than otherwise. This is clearly reflected in Fig 2F by the peak at ~130 km, which represents the genetic distance between viruses sampled in the metro Boston and Springfield areas, but a similar pattern can be seen for other pairs of metro areas. That is, viral genetics showed that SARS-CoV-2 moved more quickly between metro areas than it did to the primarily rural areas between them. This effect is detectable for genomes differing by up to 12 substitutions; viruses more distantly related than this are uniformly distributed throughout the state. Using a simple model to translate this into time, we can treat the two viruses being compared as descending from a recent common ancestor, with each descendant lineage accumulating ~0.5 mutations per generation. Thus a difference of 12 substitutions would accumulate in ~12 generations or ~60 days, suggesting that viruses diffuse throughout the entire state in about 2 months.

Enrichment for closely related viruses was highest for viruses from the same municipality (treated here as a distance of 0 km), which were 5.9x more likely to be closely related than randomly selected viruses (Fig 2G). This enrichment was more pronounced for more rural communities: within-municipality viruses were 11.3x as likely to be closely related in developing suburbs and rural municipalities, but only 5.6x in the inner core, regional urban centers, and mature suburbs (Fig 2G). Contributors to higher relatedness in less populous towns could include smaller viral population size and the greater isolation from intrastate movement noted above.

### Distinct transmission patterns between demographics

Beyond geography, the varied demographics and environments represented by the testing sectors in our dataset allowed us to look at the impact of these aspects on viral transmission. We first examined a previous finding, that BA.1 cases increased earlier and faster in colleges than in statewide data, suggesting that surveillance in colleges could provide early warnings of rising variants^16^. A possible contributor to this observation was that colleges routinely conducted asymptomatic surveillance testing, enriching for early detection of cases^16,17^. To explore this possibility, we grouped samples from lineages with enough data (BA.1*, BA.2*, BA.2.12.1*, and BA.5*) into 5 categories by setting: 1) colleges; 2) schools, workplaces, and SNFs (combined to have sufficient data to fit a regression); 3) tertiary hospitals; 4) clinics and community hospitals; and 5) public testing (Sup Fig 6). The sectors in the first two categories performed predominantly asymptomatic surveillance testing, while the remaining categories included different mixes of symptomatic and asymptomatic testing.

**Figure 3.**
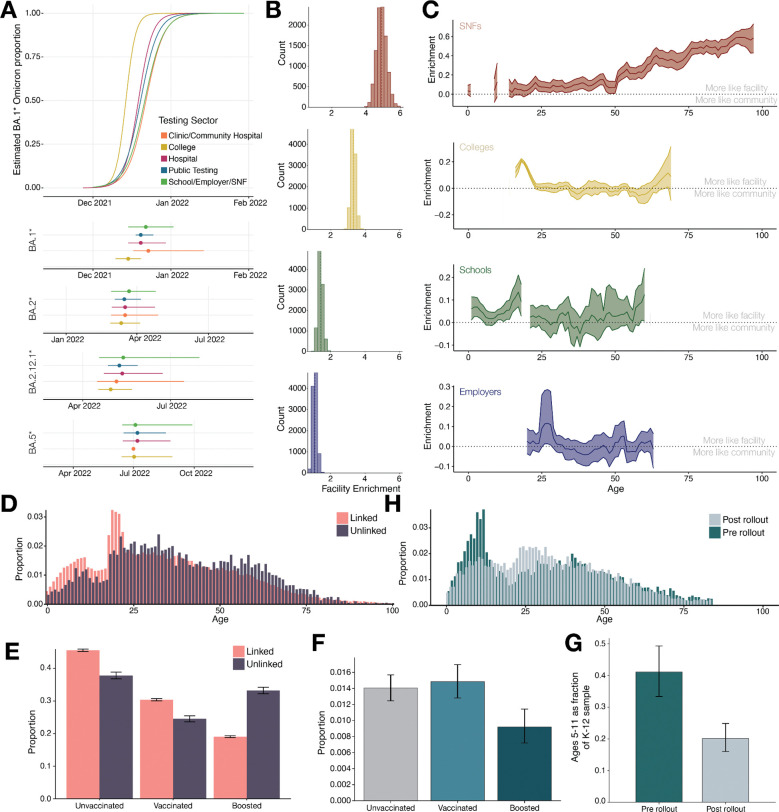
Patterns in viral relatedness and transmission across facilities and subpopulations. A) Logistic regression (top) of BA.1 and 95% confidence interval of 50% dates of BA.2, BA.2.12.1, BA.4, and BA.5. B) Histogram of the bootstrap samples of times-increase enrichment (x-axis) of closely related viruses within a facility versus among a municipality, SNFs shown in red, colleges in yellow, schools in green, and employers in blue; corresponding dashed colored line represents the sector average. C) Plot of relative enrichment (diffined as the difference in likelihood of enrichment between the facility and public testing in the case’s municipality) by age within sectors, 3-year rolling average is plotted for age groups with more than 30 samples, bootstrapped 95% confidence intervals shown around mean estimate. D) Histogram of the frequency of ages among linked (coral) and unlinked (dark purple) cases in our complete dataset; this distribution of ages further supports the enrichment findings from our testing sectors, that younger individuals are enriched due to transmission in schools and colleges and older individuals are linked due to transmission in SNFs. E) Barplot comparing the proportion of linked (coral) and unlinked (dark purple) cases among unvaccinated, fully vaccinated, and boosted individuals in our complete dataset, comparing individuals that had no closely related viruses in our data (unlinked) to those with any closely related viruses (linked). F) Barplot comparing the proportion of individuals with a descendant as detected by shared iSNVs among unvaccinated, fully vaccinated, and boosted individuals in public testing. G) The proportion of individuals aged 5–11 years among school testing in November 2021 versus in late December 2021 and early January 2022. H) Histogram of the frequency of ages among public testing data in November 2021 (blue) versus in late December 2021 and early January 2022 (gray).

The time it took for new variants to become established in our data was consistent with the previous result, with 3 out of 4 lineages reaching 50% frequency earlier in colleges by 4 to 13 days than in all other sectors (Fig 3A). That is, the early rise of new variants in colleges was not observed in other sectors also carrying out asymptomatic surveillance, suggesting that unique aspects of the college population or environment drove the rapid growth, and reinforcing the potential of colleges as settings for early viral detection. The exception to this pattern was BA.5*, which showed similar timing in all sectors (Fig 3A) but which is still consistent with this conclusion, since it rose to prominence at a time (June 2022) when colleges were not in session. For the lineages for which we had enough data (BA.1*, BA.2*, and BA.2.12.1*), we further compared college-aged individuals (18 – 22 year olds) in college testing and in public testing to all-age public testing. BA.1* reached 50% frequency in 18 – 22 year olds in colleges considerably earlier than the other settings, whereas BA.2* and BA.2.12.1* reached 50% frequency earlier in 18 – 22 year olds in both colleges and public testing than in all-age public testing (Sup Fig 7). This suggests that both the college environment and the 18 – 22 age group have unique features that contribute to early viral transmission and may warrant enhanced surveillance.

We next explored transmission within close-contact settings, represented in our dataset by colleges, schools, SNFs, and employers, looking specifically at the relative importance of within-facility transmission compared to transmission with the surrounding community. As a quantitative measure, we calculated the enrichment in the likelihood of closely-related viruses within each facility compared to viruses collected in public testing from the same municipality, which is a proxy for how likely viruses are to be part of the same local transmission network in the two settings. Not surprisingly, the likelihood of close relatives in the same facility on average was 12.9 percentage points higher (12.1 – 13.8%, within-facility: 21.3%, 20.7 – 21.9%; within-community: 8.4%, 7.7 – 9.1%) representing a 2.7-times (CI: 2.5 – 2.9x) enrichment of closely related viruses within facilities compared to public testing within municipalities. More interesting was the large variation across facility types: SNFs were enriched 4.9-times (CI: 4.3 – 5.5x), while colleges and schools showed lower enrichment (3.3x, CI: 3.1 – 3.6x and 1.4x, CI: 1.2 – 1.7x respectively), and employers showed no significant enrichment (1.1x, CI: 0.9 – 1.3x) (Fig 3B). To test if differences in sampling densities across sectors could explain these results, we performed these analyses on subsampled sets of genomes within each sector and found that the overall trends in enrichment were consistent.

In several facility types, distinct subpopulations could be identified and analyzed separately because their age distributions had limited overlap (staff vs. students in schools and colleges, staff vs. residents in SNFs). For these facilities, we carried out the same within-facility to within-municipality comparison, but now broken out by age. We found relatively little enrichment of closely related viruses among staff-aged individuals within schools (>30 years), colleges (>30 years), and SNFs (<50 years), suggesting that workplace exposure played a relatively small role in their infection risk. In contrast, the subpopulations that the staff were serving showed substantial enrichment of closely related viruses within facilities. In SNFs, enrichment increased sharply with age, beginning at 50 years old, with the oldest individuals (90+ years) showing ~60% enrichment. In colleges and schools, student-aged individuals (~19 years and ~17 years respectively) showed the highest enrichment (~20% and ~10% respectively) indicating more connection to their school environment than for staff (Fig 3C). While these results show greater relative involvement in community transmission networks for staff than for non-staff, they are not informative about the cause. It is possible that transmission between staff and non-staff (in either direction) was less common than that within non-staff, or that staff were more involved in transmission outside of the facility than non-staff, or most likely some combination of both effects.

### Vaccination was qualitatively associated with decreased infection and transmission

We next explored whether distinct subpopulations were disproportionately responsible for transmission, using two genomic approaches. The first sought to distinguish individuals who transmitted the virus from those who did not, using as a proxy those who had a virus with a close relative elsewhere in the dataset (“linked”) from those that did not (“unlinked”). The second approach looked for evidence that a specific individual transmitted the virus at least once, by identifying those who carried within-host viral variants that were seen later in other individuals at consensus level, suggesting that they were the source of the variants^18^. That is, the first approach identifies members of the same local transmission network while the second seeks to identify direct descendants of a particular infected individual.

Both approaches suggest that younger age and male gender contribute to a higher probability of initiating a transmission. Using the first approach, we found that unlinked individuals were significantly older (p < 0.001 by Mann-Whitney U test), with a median age of 36 years (IQR: 23 – 54) for unlinked versus 31 years (IQR 19 – 48) for linked (Fig 3D). There was a similar trend toward lower transmission with increasing age in the second approach but it was not statistically significant (Sup Fig 8F). While gender proportions were the same between linked and unlinked individuals, the second approach showed a slightly higher probability (1.21x, 95% CI: 1.02x - 1.41x) for males to initiate a transmission (Sup Fig 8A, 8B). Neither method provided evidence for an association between the presence of symptoms and transmission, but this may be due to artifacts introduced through reporting of symptoms (Sup Fig 8C, 8D).

Applying the same approaches to the relationship between transmission and vaccination, we found that having received a booster dose of a COVID-19 vaccine was correlated with lower likelihood of transmission. Unlinked individuals in our entire dataset were significantly more likely to have been boosted, with 33.2% (32.3 – 34.3%) of unlinked individuals being boosted compared to 19.1% (18.8 – 19.4%) of linked individuals (Fig 3E). Similarly, the second approach showed that boosted individuals were only 0.65x (0.50x - 0.84x) as likely as unvaccinated individuals to initiate at least one transmission event (Fig 3F). However, no significant difference was found between unvaccinated and vaccinated but not boosted individuals.

To assess whether the difference between boosted individuals and those who were vaccinated but not boosted was caused by the shorter time since the most recent vaccination for the former, we also assessed whether individuals were less likely to transmit the virus when they had received their initial vaccination recently. We compared individuals who were vaccinated early in the initial vaccine rollout to those who received their primary vaccination coinciding with the availability of booster doses. We observed a concordant trend where individuals vaccinated early (within the first 6 months of vaccine availability) and late (during the period of booster rollout) were 1.04x (0.80x - 1.36x) and 0.85x (0.54x - 1.33x) as likely to initiate at least one transmission event as unvaccinated individuals, respectively (Sup Fig 8E), suggesting that more recent vaccination may decrease the probability of transmission. Although not significant, this trend is consistent with previous observations of the waning effect of vaccination on SARS-CoV-2 infection and transmission^19^. However, diagnostic PCR Ct values do not differ with vaccination, suggesting a potential role of behavior rather than biology (Sup Fig 3).

Estimating the effect of vaccination on infection was not possible with this passively collected dataset, but we were able to observe the qualitative impact of vaccination on infection in one cohort, 5- to 11-year-olds. In the US, this group first became eligible for COVID-19 vaccination on October 29, 2021, two days before the beginning of our study, with administration of first doses ramping up in mid-November (Sup Fig 9A) and second doses following in mid-December. We were therefore able to compare age distributions of infected individuals immediately before and after the acquisition of vaccine-mediated immunity in this cohort. The proportion of individuals in this age group among samples from the school testing sector decreased by 51% between the earlier period (November) and the later one (late December/early January) (Fig 3G). To address whether this decrease reflected real changes to the infection rate for 5–11 year olds (rather than changes in school testing during the winter holiday period, for example), we examined 5- to 11-year-olds in the public testing sector for the same two time periods. We again saw a marked decrease in cases from this cohort in late December/early January, which had in November been the most over-represented age group (Fig 3H). The concordance of school testing and public testing suggests that vaccination provided considerable short-term protection against infection; more detailed analysis (Sup Fig 9B) suggests that the protection applied to both Delta and BA.1*, the dominant lineage during the later time period.

### Sampling rates needed for detection of novel lineages

A practical question that arose as the COVID-19 pandemic progressed was the scale of genomic sequencing required to achieve the basic surveillance aims of detecting and monitoring new variants. For example, the US CDC’s National SARS-CoV-2 Strain Surveillance program had a target of sequencing ≥5% of confirmed cases, with the intent being to detect new lineages while they were at ≤1% frequency in the local population^20,21^, but this target became difficult to interpret when COVID-19 testing largely shifted to at-home tests, reducing PCR test results reported into public health surveillance systems. Previous efforts to understand how much sequencing was needed to detect lineages before they reached ≥1% frequency have relied on simulations^22^; the scale of our dataset, however, allowed us to empirically investigate how different sequencing rates–in terms of total genomes rather than a percentage of confirmed cases–affected the ability to detect and characterize the growth of new lineages.

**Figure 4.**
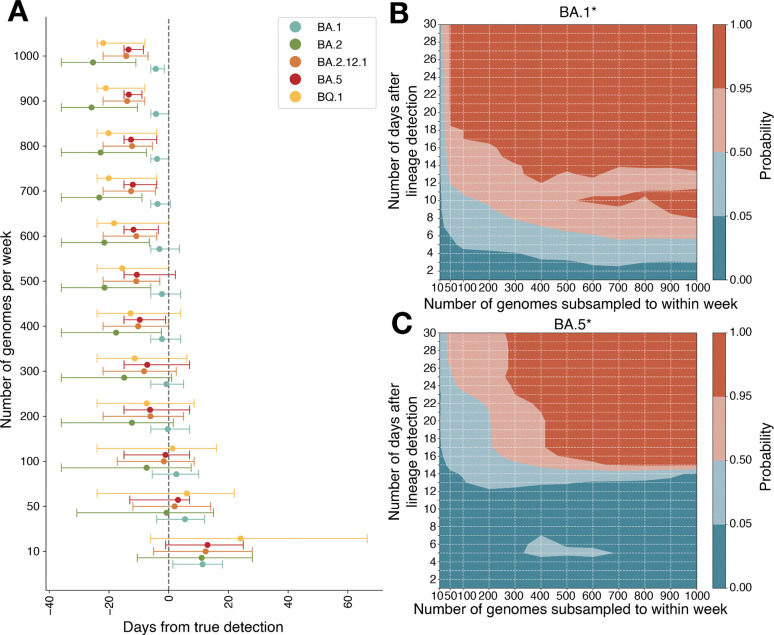
Detection of novel lineage under different sampling rates. A) Scatter plot with 95% credible intervals for the probability that a variant would be detected at any frequency with only N genomes per week. The full dataset was subsampled 1000 times randomly to each sequencing rate. The black dashed line is the true date of detection within the entire dataset. B) Subsampling BA.1* empirical data to N genomes per week, fitting a logistic regression starting on 1 day after BA.1* reached 3% frequency in the dataset. Plotted is the probability that the 95% CI of the growth rate is greater than 0, indicating logistic growth. Red colors indicate greater than 50% probability. C) Subsampling BA.5* empirical data to N genomes per week, fitting a logistic regression starting on 1 day after BA.5* reached 3% frequency in the dataset. Plotted is the probability that the 95% CI of the growth rate is greater than 0, indicating logistic growth. Red colors indicate greater than 50% probability.

To understand the rate of sequencing needed to detect the arrival of previously identified lineages, we estimated the probability of detection at a given weekly sequencing rate, based on a binomial distribution parameterized with variant frequencies estimated from our complete dataset. We simulated different sequencing rates 1,000 times per lineage (BA.1*, BA.2*, BA.2.12.1*, BA.5*, and BQ.1*, the lineages for which there was enough data to analyze) to assess how sampling rate would influence time of first detection, relative to the observed date each variant reached 1% prevalence (the threshold used by the CDC estimates^20,21^) in the complete dataset. Our results indicate that sequencing 100 genomes per week would lead to detecting a new variant before it reached 1% frequency more than 50% of the time, with the probability increasing with increasing sequencing rate until 500 genomes per week; at this rate, four of the five lineages (BA.2*, BA.2.12.1*, BA.5*, and BQ.1*) had >95% probability of being detected by the target date, with little gain from even higher sampling (Fig 4A). BA.1*, on the other hand, rose rapidly enough in the population that any sampling rate would not have provided earlier detection relative to when it reached 1% frequency. We also calculated the probability of detecting growing lineages using PhyloSamp^22^, a calculation tool based on binomial sampling theory. We found consistent results (50% probability of detection before 1% prevalence with 100 genomes per week and 95% probability with 420 samples per week; Sup Fig 10).

Beyond initial detection, an important goal of genomic surveillance is to identify lineages that have rapid growth rates. We evaluated whether, as a function of sampling rate, we could confidently determine that the relative proportion of a given lineage (previously known or not) in the total viral population was increasing. We subsampled the complete dataset at rates of 10, 50, 100, 200, up to 1,000 genomes per week, repeating this process 100 times. Within each subsample, we fit a logistic regression starting from the day the lineage (BA.1*, BA.2*, BA.2.12.1*, BA.5*, and BQ.1*) reached 3% frequency and extended the time span of the regression by 1 day for 30 days afterwards, recording the date at which the growth rate of each lineage became positive with 95% confidence. Here, we use 3% frequency to minimize the highly stochastic nature of lineages that were maintained at low frequencies before growing exponentially. As expected, we found that fast-growing variants like BA.1* required fewer genomes to detect population increases: based on our empirical data, 300 genomes per week were sufficient to determine within 14 days that BA.1* was growing (Fig 4B), while slower-growing variants like BA.5* would have required at least 400 genomes per week to detect growth within 21 days (Fig 4C). Overall, across both metrics, sequencing 500 genomes per week, which translates to a rate of roughly 7.1 genomes per 100,000 people, would capture both the earliest detected arrival and the growth of a lineage. While these estimates are based on surveillance from Massachusetts that occurred during an intense public health emergency with a heightened level of surveillance, answering specific questions (e.g. detection of the arrival of a variant of concern) in other contexts may require fewer genomes. For example, as few as 50 genomes per week in Massachusetts would have had similar detection times of new lineages.

## Discussion

Large scale genomic surveillance provided valuable insights into the emergence and spread of SARS-CoV-2 variants during the COVID-19 pandemic, but many analyses were necessarily hindered by a lack of epidemiological metadata associated with the viral genomes. Here we leverage high resolution SARS-CoV-2 genomic surveillance and matched epidemiological metadata across multiple variant waves to explore patterns of viral spread within and between communities, differing transmission dynamics within subpopulations, and individualized risk of transmission and infection.

The arrival of multiple new lineages during our study period, each effectively a new outbreak, allowed us to explore patterns in where lineages arrived, where they subsequently spread, and how they behaved across different populations and settings. The picture that emerges is one in which major urban areas played the most important role in the arrival of new lineages, with introductions from outside the state representing a larger fraction of cases in urban areas than elsewhere. Subsequent spread was fastest to adjacent suburbs, followed by spread between regional centers in the state, while spread to less populated areas was slower, taking roughly 2 months for new lineages to be uniformly distributed throughout the state. Our data suggests the most effective avenues to reduce transmission may differ by geographic density—for example, limiting new introductions into urban centers and breaking local transmission chains in rural areas.

With knowledge of the facility types from which the genomes were obtained, we were able to assess the growth and transmission of these lineages in different subpopulations. Young adults, especially those in colleges, were leading indicators for the establishment of new lineages; that is, lineages grew within young adults before the rest of the state, with this effect being strongest for young adults in college settings. This suggests the potential value of enhanced surveillance in college settings, which can serve as early indicators of emerging lineages. Perhaps unsurprisingly, high contact environments like schools, colleges, and SNFs showed high levels of transmission within their local populations, but the effect showed considerable heterogeneity not only between different kinds of facilities, with SNFs having the largest enrichment for local transmission, but between subpopulations in the same facility. Consistently, staff-aged individuals were more connected to outside communities than the students or residents that they served.

It was difficult to quantify the effect of vaccination on infection, as we were unable to determine vaccination rates in those not infected. Nevertheless, we were able to see evidence for a short-term protective effect against infection from vaccination: the rollout of vaccination for 5–11-year-olds took place during our study period, allowing us to observe an immediate decrease in infections in this age group, both in within-school surveillance testing and in broader public testing. The association between vaccination and transmission, on the other hand, was easier to assess, since transmission detection depended on comparing descendants of viruses in vaccinated and unvaccinated individuals, which could be done based entirely on the genetics of samples within our dataset. Those who received more recent and additional vaccine doses, including booster doses, had decreased probability of initiating transmission. To what extent this represented a causal effect of vaccination, rather than differences in behavior or other risk factors is impossible to determine, however.

The challenges we faced in these analyses are common in studies of COVID-19, stemming both from the nature of the dataset, which is observational and based on convenience sampling, and from the complex nature of the pandemic, in which the immunological landscape changed with ongoing infection and vaccination, the testing and sampling landscape changed with the transition to primarily home diagnosis, and the urgency of the public health emergency took priority over perfect record-keeping. Added to these challenges is the difficulty in distinguishing biological from behavioral effects; e.g. vaccinated individuals are likely to have a different exposure profile than the unvaccinated. Nevertheless, this study illustrates that the combination of genomic and demographic data can yield a much more detailed understanding of the complexity of transmission patterns as they unfold across different geographic scales than either type of data alone.

## Methods

### Ethical approvals

As previously described^18^, the research project (Protocol #1603078) was reviewed and approved by the Massachusetts Department of Public Health (MADPH) Institutional Review Board and covered by a reliance agreement at the Broad Institute. An additional non-human subjects research determination was made by the Harvard Longwood Campus Institutional Review Board.

### SARS-CoV-2 RT-qPCR

For specimens submitted for testing under Emergency Use Authorization to the Broad Institute’s CLIA-certified laboratory, Clinical Research Sequencing Platform (now Broad Clinical Labs), total RNA was extracted from inactivated anterior nasal (AN) swabs using the Thermo Fisher MagMAX Viral RNA Isolation kit and presence of virus was confirmed by RT-qPCR assay detecting the N1 and N2 SARS-CoV-2 gene regions. Ct values for the N1 gene were used to compare viral titers between individuals; samples for which the RP positive control gene had a Ct>32 were excluded from the analyses to prevent biasing from poorly collected samples. A specimen was determined positive if the initial cycle threshold of the N1 probe was less than or equal to Ct 40. For the purpose of library construction, a lower Ct value threshold of 30 was chosen based on the higher influx of positive samples available.

### SARS-CoV-2 sequencing

Candidate samples were re-extracted from the source material for Illumina sequencing. Libraries were prepared using the NEBNext ARTIC SARS-CoV-2 FS Library Prep Kit and transitioned through ARTIC, VarSkip Short v1, v1a, or v2a amplicon primers to ensure genomic coverage of emerging variants. During library preparation, some volumes were adjusted from manufacturer recommendations to accommodate 384-well plate reactions and high-throughput automated processing. Libraries were sequenced on Novaseq SP flowcells with 75-nucleotide paired-end reads.

### Genome assembly and analysis

For genomes generated at the Broad Institute, we conducted all analyses using viral-ngs 2.1.28 on the Terra platform (app.terra.bio). All of the workflows named below are publicly available via the Dockstore Tool Registry Service (dockstore.org/organizations/BroadInstitute/collections/pgs). Briefly, samples were demultiplexed, reads were filtered for known sequencing contaminants, and SARS-CoV-2 reads were assembled using a reference-based assembly approach with the SARS-CoV-2 isolate Wuhan-Hu-1 reference genome NC_045512.2 (using workflow sarscov2_illumina_full.wdl). We processed all raw read data using a reference-based consensus calling method with the same NC_045512.2 reference genome. The workflow is publicly available on GitHub. Assembled genomes meeting the CDC criteria for submission to public repositories (unambiguous length ≥24,000 nt and successful gene annotation) were deposited in NCBI Genbank and GISAID immediately upon completion. Raw reads for all samples (including those that did not produce a successful genome) were deposited in NCBI SRA. All NCBI data were deposited under BioProject PRJNA715749.

We used LoFreq version 2.1.5 to call intrahost single nucleotide variants (iSNVs) with default parameters (minimum read depth ≥10, strand bias <85%, and default iSNV quality scoring)^23^. Variants with frequency ≥3% and in positions with read depth ≥100 were used for downstream analysis. Minor variants arising in primer binding sites were masked^24^. All non-fixed consensus mutations (<97% and >50%) were flipped to their corresponding minor variant (e.g. a mutation at C11803T at 65%, would now show up as a T11803C at 35%). An additional filtering step was applied to account for sequencing bias^25,26^. Briefly, a binomial distribution was applied that took into account the total number of variant reads, sequencing depth, and sequencing error rate. Variants with significance (≤0.05) after applying a Bernoulli correction were kept for downstream analysis^26^.

### Phylogenetic tree construction

All genomes paired with metadata during the study period (November 1, 2021, to January 17, 2023) were used for phylogenetic inference (n=94,404). Prior to phylogenetic inference, genomes were aligned using Nextclade CLI version 1.3.0. Genomes were excluded if they 1) contained any mixed bases, 2) were missing >10% of the SARS-CoV-2 genome, 3) lacked adequate metadata, or 4) were denoted as “bad” during Nextclade QC. The remaining (n=91,080) genomes were masked using a light masking scheme^27^.

To understand the number of introductions into each municipality, we generated 60 variant-specific (10 per variant) maximum likelihood phylogenetic trees using publicly available SARS-CoV-2 genomes from Genbank (retrieved January 23, 2024). Datasets were filtered to only include genomes collected prior to the date that each major lineage reached 50% frequency in MA. The resulting dataset was further subsampled so that 1% of confirmed infections were sequenced within each geographic resolution (country, state, municipality), as the geographic distribution of cases in Massachusetts was skewed towards highly urban areas. We constructed a maximum-likelihood (ML) phylogenetic tree using FastTree^28^ with 100 bootstraps and otherwise default parameters.

To understand the timing of introductions, UShER was used to select the 5 closest phylogenetic neighbors for each MA genome. An additional 20 and 10 genomes per week were additionally added from a USA and global dataset, respectively. The resulting dataset was used to generate an ML phylogenetic tree for each major lineage using FastTree^28^ with 1000 bootstraps and otherwise default parameters. We analyzed the resulting phylogenetic tree along with 100 randomly selected bootstraps.

To unlink sample size, we generated two sets of equal temporal subsampled trees. First, using the augur filter tool, we took 100 randomly selected genomes per municipality type. An additional randomly sampled 2000 genomes per month were taken from the USA and global dataset, with a focus on genetic proximity to our dataset samples. Second, we took 5 genomes per week from each municipality in Massachusetts that had a weekly average sequencing rate of 5 genomes per week. Again, we took an additional randomly sampled 2000 genomes per month from the USA and global dataset, with a focus on genetic proximity to our dataset samples.

Using custom scripts, the resulting phylogenies were processed using the refine, ancestral, traits, and export steps of the Nextstrain pipeline^29^, then visualized using baltic^30^.

### Viral introductions

To identify viral introductions into Massachusetts municipalities, we assigned a trait to each genome in the phylogeny of either “out-of-state” or the name of the municipality. We used Nextstrain’s ancestral inference^29^ to infer the state of that trait for each internal node of the tree. We defined an introduction using baltic^31^ as instances of ancestral nodes on the branch inferred to be “out-of-state” with confidence ≥70% and a municipality descendant node.

For viral movements occurring between Massachusetts municipalities, we applied the same logic, defining a viral movement between municipalities as instances of an ancestral node and descendant node being different municipalities, both with confidence ≥70%. We combined the movement data between origin and destination municipalities with associated variables such as case counts, percent positivity, and population size and density for both origin and destination. Movements across the 10 replicate phylogenies for each variant were deduplicated on the origin, destination, date, and downstream genomes. The remaining movements were then counted based on origin, destination, and date. We used a Generalized Linear Model (GLM) with a Gaussian family and identity link function to examine the relationship between movement counts and various predictors. The model was specified as:

Movement_count∼β0+β1(predictors)+ϵ

Model fitting was performed using the Iteratively Reweighted Least Squares (IRLS) method in the statsmodels library v0.14.2 ^32^ in python. Model performance was assessed using the Akaike Information Criterion (AIC) and log-likelihood.

### Normalized age distribution

The age distribution was calculated for all public testing samples with submittable genomes and recorded subject ages during two periods, November 1, 2021 – November 30, 2021 and December 15, 2021 – January 13, 2022. The statewide population by age was interpolated using a cubic spline from US Census estimated data for 2021 (https://www.census.gov/data/tables/time-series/demo/popest/2020s-state-detail.html).

### Pairwise analysis

First, all possible pairs of genomes within the same lineage, collected within 10 days of each other, and collected in the public testing sector were identified. Then, the genetic distance between all possible pairs was calculated using the ape^33^ package v5.7.1 in R. Additionally, we similarly calculated all pairs among all genomes collected at SNFs, employers, schools, and colleges. We calculated geographic distance with the R package *geosphere*^34^ V 1.5.18 as the haversine distance between the interior points of each pair of municipalities.

### Identification of closely related pairs

The identification of closely related cases was performed in Julia (v1.9). To identify all pairs of closely related cases within our dataset, first a random case was selected and all cases occurring within 10 days of this case were identified. Then the genetic distance from the initial randomly selected case to all of these temporally linked cases was calculated, and cases with a genetic distance of 2 or fewer SNPs were recorded as being closely related. This process was repeated until all cases had been compared against all other temporally linked cases. Resulting cases which had any number of additional closely related cases were then recorded as being ‘linked’ while cases with no additional closely related cases were ‘unlinked’.

To compute the percent enrichment of closely related viruses within the same testing facility as compared to the same municipality we calculated, per virus, the percent of contemporary (occurring within 10 days) facility cases which were closely related and subtracted the percent of contemporary (occurring within 10 days) municipality cases collected in public testing which were closely related. To additionally get a relative percent change, the likelihood of close relatives in each facility was calculated on a sector level (that is, summing the number of related cases for all cases within a given sector to avoid dividing by zero) and then divided by the percent of contemporary closely related in public testing in the community. Due to high variability in the likelihood of close relatives among public testing for specific age groups in each sector, we only calculated this on average for sectors, not within specific ages in each sector. We then averaged all cases per sector to report summary findings. To describe these patterns over age groups we computed overlapping, 3-year rolling averages per sector. Age groups which had fewer than 30 viruses for comparison were dropped from the analysis.

### Detection of putative transmission events

An individual was defined as a transmitter when an index and contact met the following criteria: 1) the index and contact consensus sequences differed by no more than two consensus-level changes, 2) the consensus level changes in the contact were detected at sub-consensus levels in the index, 3) the contact appeared downstream of the index in a divergence phylogeny, 4) the collection date of the index was ≤6 days before that of the contact, and 5) if two genomes were identical, the defining mutations were at ≤90% frequency in the index, only seen <5 times in the community, and rose to 100% frequency in the contact.

### Estimation of sequencing rates to detect novel lineages

A multinomial regression model was applied to estimate the daily frequencies of variants BA.1*, BA.2*, BA.2.12.1*, BA.5*, and BQ.1*. Using the estimated variant frequencies, a simple model was used to estimate the earliest possible detection dates. This model incorporates a binomial draw, accounting for both sampling rates and the estimated frequencies of each variant, outputting the number of variant genomes we would detect on a given day. This model was used to calculate the probability of detecting a variant genome: this model was run 1,000 times per genome sequencing rate (10, 50, 100, 200, up to 1,000 genomes per week). The resulting probabilities were used to estimate a 95% credible interval of the first date of detection for each variant under each sequencing rate.

We additionally applied the vartrack_prob_detect_cont function from PhyloSampe to verify these modeling results. For this function we set the coefficient of detection ratio to 1 (i.e. assuming no difference in the probability of detection between lineages), the initial prevalence to 0.0001, and the percent of samples which generate high quality genomes to 100% (to reflect that the modelling approach we employed relied on the number of high quality genomes). This function was then run with logistic growth rates of 0.075, 0.125, 0.175, and 0.225 and sampling rates ranging from 7 genomes per week to 1000 genomes per week.

### Estimation of sequencing rates to detect growth of novel lineages

To estimate how many genomes would be needed and when growth of variants would be detected after reaching 3% frequency, the dataset was subsampled 100 times at rates of 10, 50, 100, 200, up to 1,000 genomes per week. For each subsample, the date when the lineage reached 3% frequency was recorded. A logistic regression was generated starting from day 1 (after reaching 3% frequency) through 30 days, capturing the day when the growth rate became positive with 95% probability. The probability was plotted that a positive 95% growth rate could be detected within each sampling rate N days after the lineage reached 3% frequency.

### Statistical comparison of 50% date

A logistic growth model was used to analyze the growth trajectories of Omicron sublineages (BA.1*, BA.2*, BA.2.12.1*, and BA.5*) in each sector. For each sector, the logistic growth model was fit using nonlinear least squares (NLS) regression to estimate the parameters growth rate and lineage frequency. The model for each lineage was constrained to only consider data starting from the first day where the proportion exceeded 0. The fitted models were used to generate predicted frequency of each lineage over time.

The tsum.test() function within BSDA^35^ was used to compare the growth rates of the variants between sectors. Specifically, the significance was tested of the differences between sectors in the timing of when each variant reached 50% frequency. The tsum.test() function was used while accounting for standard errors and sample sizes. A 95% confidence level was applied to assess the significance of differences in the growth rates and 50% threshold timing.

### Statistical analysis

All statistical analyses were performed in R (The R Core team, 2017), python 3, or Julia (v1.9). Pairwise ranksum tests for comparisons of Ct values were performed using the ranksum function in scipy^36^. All multiple comparisons were corrected using multipletests in statsmodels^32^ using the Benjamini-Hochberg correction. All additional statistical details including sample numbers can be found in the figure legends.

## Supplementary Material

Supplement 1

Supplement 2
