# Supplementary material for: Geospatial and demographic patterns of SARS-CoV-2 spread in Massachusetts from over 130,000 genomes
Source: medRxiv. 2025 Apr 6:2025.04.04.25324273. Preprint. [Version 1] doi: 10.1101/2025.04.04.25324273 (PMC11998852; doi:10.1101/2025.04.04.25324273)
Supplement: Supplement 2 [file media-2.pdf]

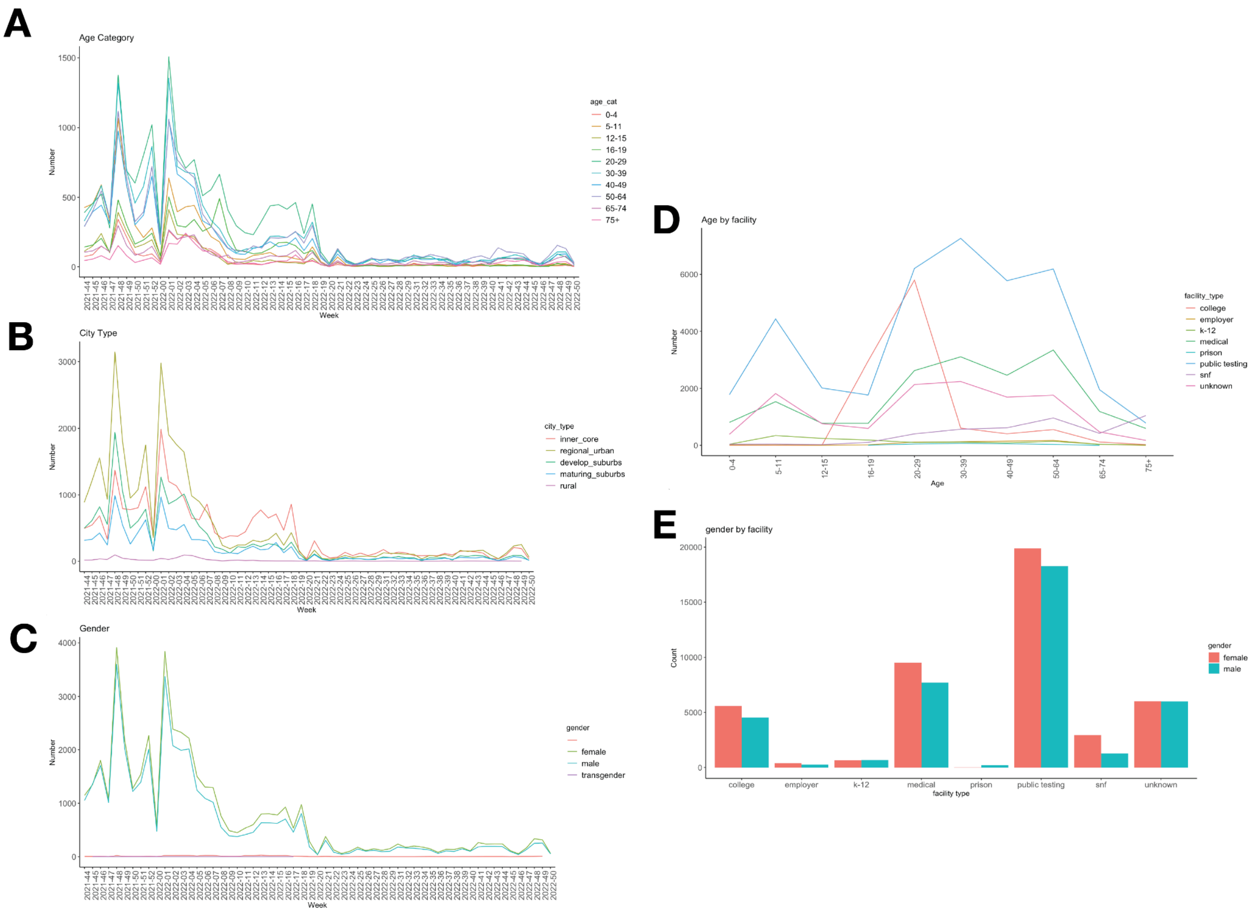

Supplemental figure 1. Temporal distribution of the number of genomes collected according to their age (A), municipality type (B), and gender (C). Number of genomes collected in each facility type by age (D) and gender (E).

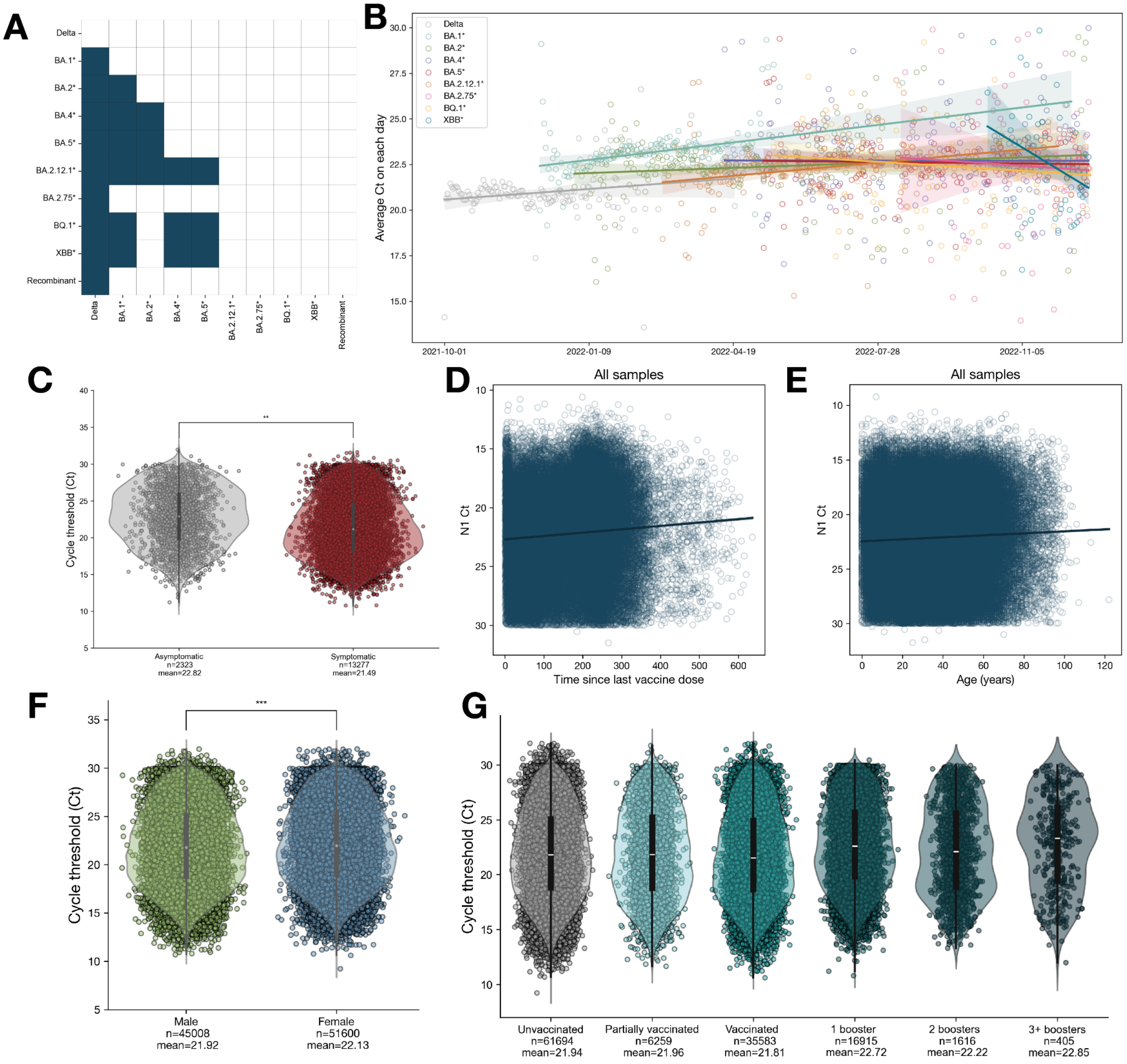

Supplemental Figure 2. Variables influencing viral load. A) Heatmap of all pairwise comparisons of viral lineage Ct. If a square is colored, it means that comparison was significantly different after multiple testing correction. B) Linear regressions of the average N1 cycle threshold (Ct) value for each lineage on each day. Ct values were markedly lower for a lineage when it was first emerging than when its prevalence was decreasing in the population, in line with previous reports where average Ct values predicted community SARS-CoV-2 case counts. Shaded regions represent 95% confidence intervals. C) A violin plot comparing Cts of symptomatic and asymptomatic. D) In a univariate analysis, we find that as time increases receiving a vaccine dose, Ct decreases by 0.002 Cts each day out from vaccine dose ($p < 2e-16$), suggesting higher viral loads with increased time from last vaccine dose. Plotted is a scatter plot with a linear regression of viral ct vs time since last vaccination. E) We find that age

has a significant association with viral load, with each year corresponding to a 0.001 decrease in Ct, though this estimate is likely not clinically significant. Plotted is a scatter plot with a linear regression of viral ct vs age. F) We find that gender has a significant difference in Cts (male mean, 21.92; female mean, 22.13), but again, likely not clinically significant. A violin plot comparing Cts of male and female individuals. G) Examining number of vaccine doses without categorizing individuals into vaccine statuses based on brands and number of doses, we find in a univariate analysis that average Cts increase by 0.14 with each vaccine dose (p<2e-16), in line with other reports that boosted individuals have higher Cts and therefore lower viral loads. Plotted is a violin plot of Cts for each vaccination status.

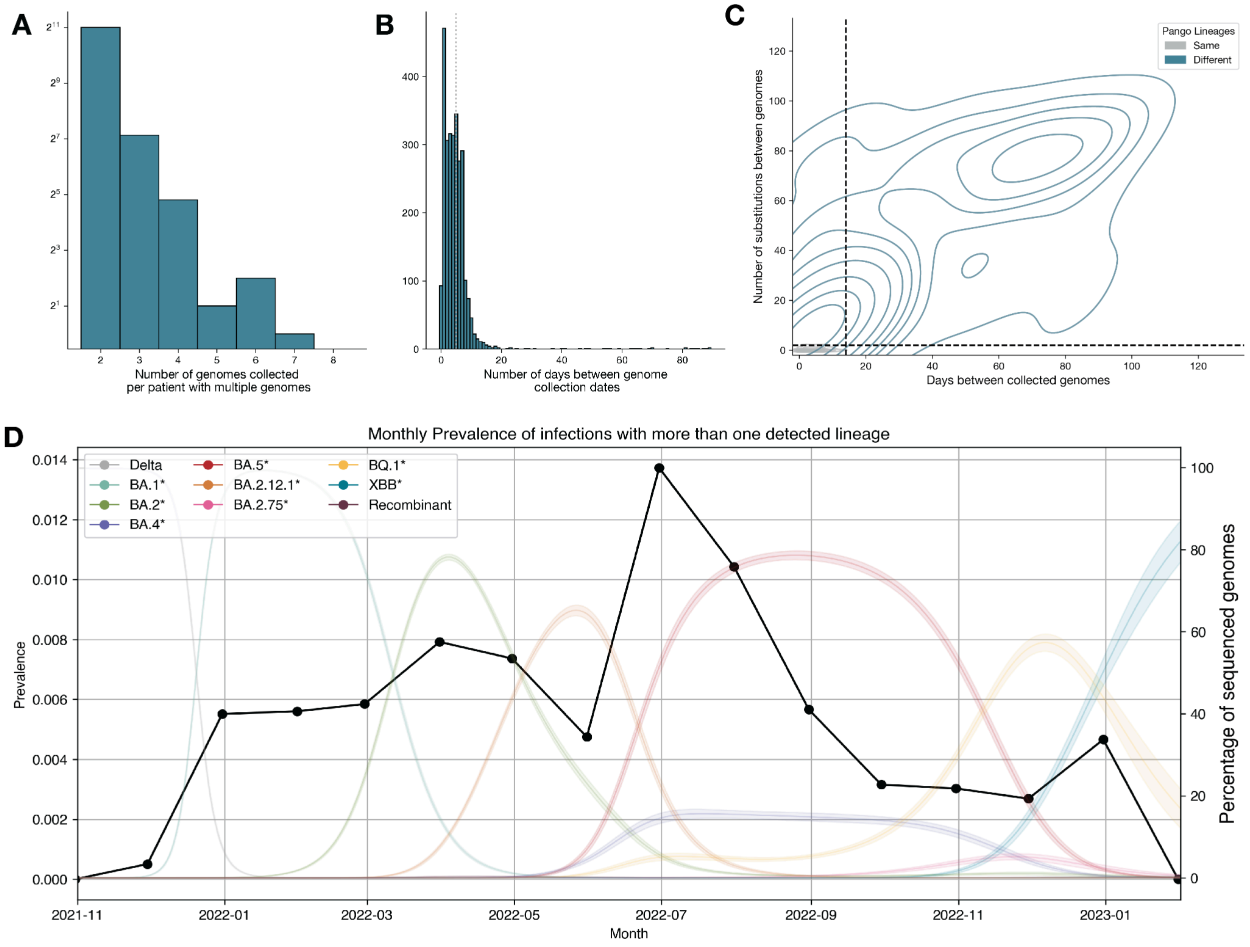

Supplemental figure 3. Individuals with more than one genome. Within our dataset, we were able to link genomes back to an individual within 90 days of their first genome. A) histogram of the number of genomes collected for individuals who had >1 genome in the dataset. B) Histogram of the number of days between genomes. The dotted line is at 4 days, which is the median number of days between genomes for individuals with multiple genomes. C) Kernel density plot of the number of days between collection and the number of mutations between samples. Individuals with multiple samples that contained the same lineage were clustered below 2 mutations and below 14 days, suggestive of the same infection. D) Plotted is

the monthly prevalence of individuals with detected mixed infections. Plotted behind is a multinomial regression of circulating lineages.

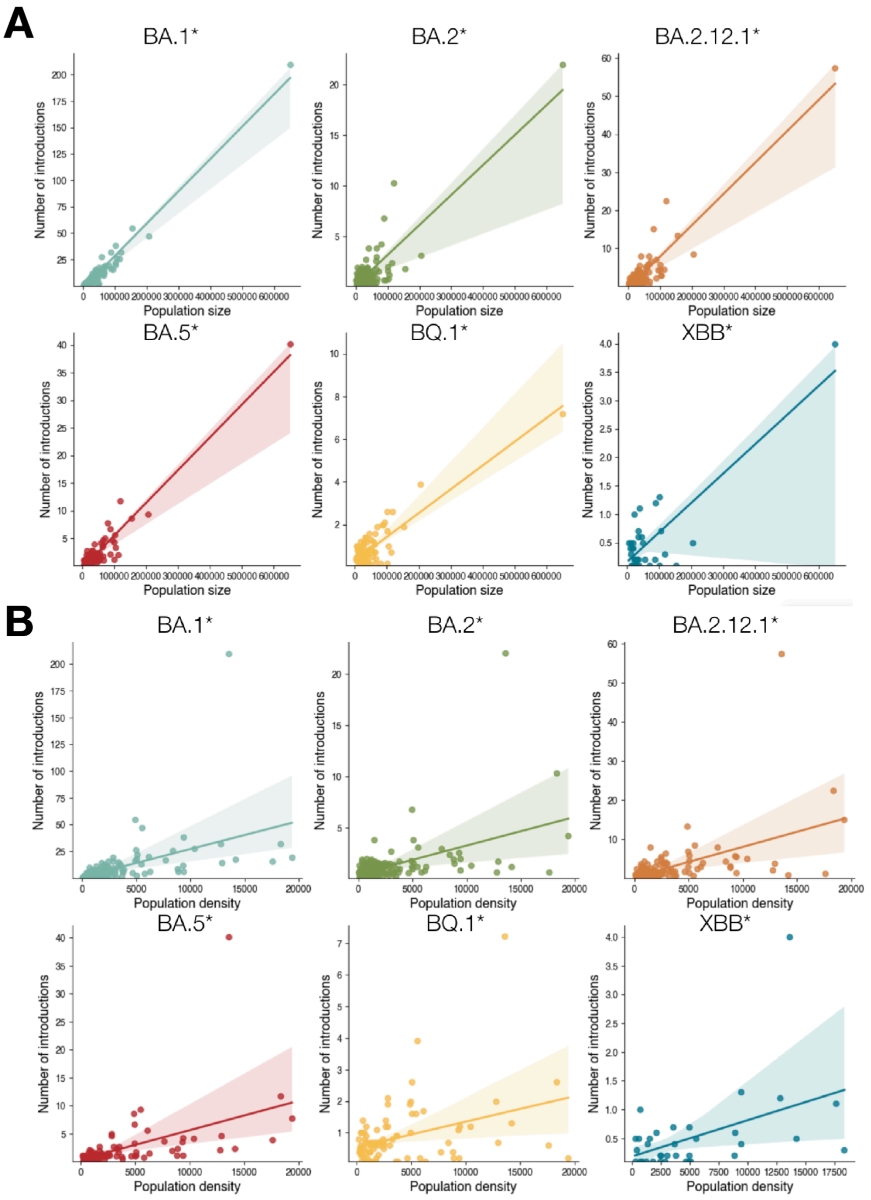

Supplemental Figure 4. Viral introductions per municipality by lineage. Datasets were filtered to only include genomes collected prior to the date that each major lineage reached 50% frequency in MA. The resulting dataset was further subsampled so that 1% of confirmed infections were sequenced within each geographic resolution (country, state, municipality), as the geographic distribution of cases in Massachusetts was skewed towards highly urban areas. A) Each scatter plot is the number of detected introductions per municipality by municipality population. Each plot is specific to a single lineage. A linear regression was fit and the shaded region represents the 95% CI. B) Each scatter plot is the number of detected introductions per

municipality by municipality population density. Each plot is specific to a single lineage. A linear regression was fit and the shaded region represents the 95% CI.

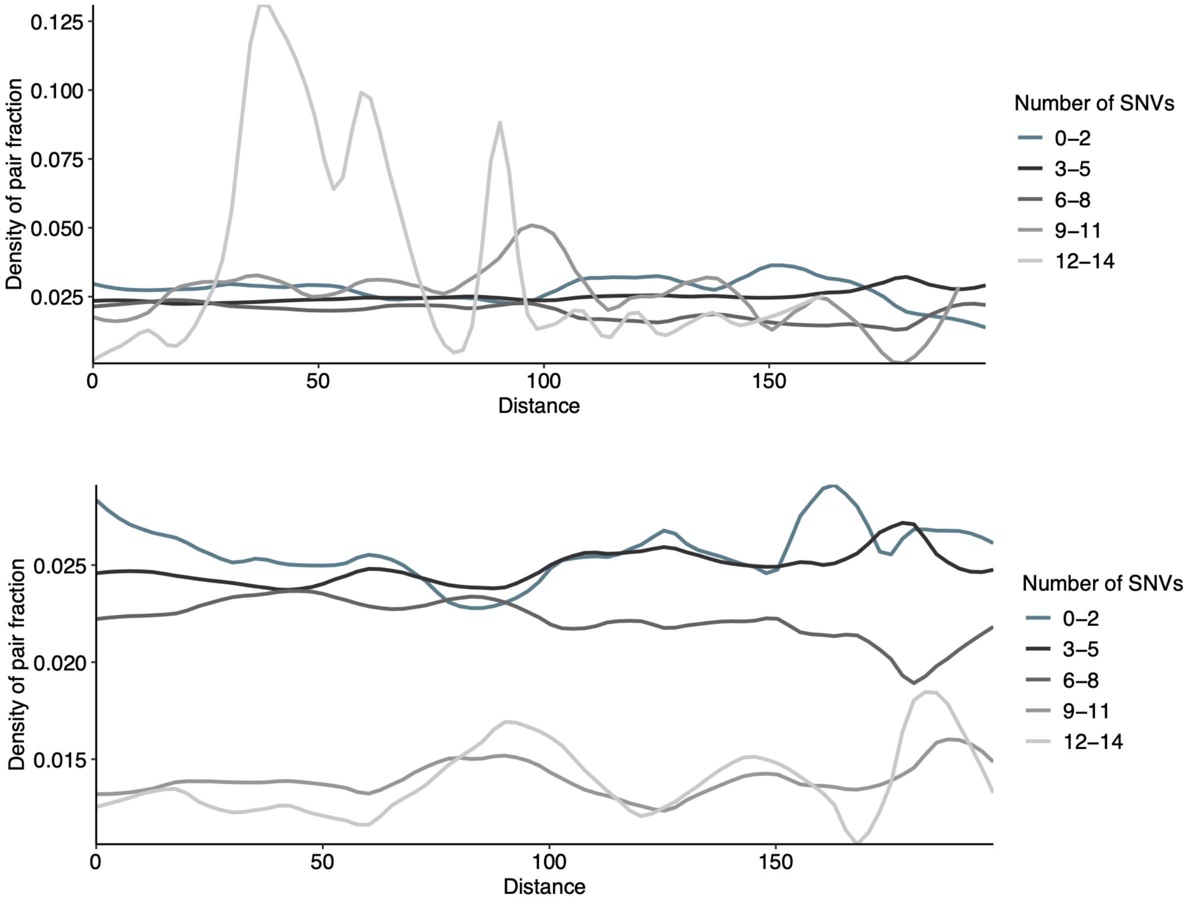

Supplemental Figure 5. BA.1 shows a lack of geographic structure as compared to other lineages. Relationship between genetic and geographic distance (as the density of the fraction of pairs of a given SNP difference) for the exponential phase of BA.1*'s emergence (top; defined as any case detected before Dec 21, 2021) and the plateau and falling phase of BA.1*'s emergence (bottom).

Testing sector	Number of genomes	Number of unique facilities
Public testing	40,888	29
College	22,477	154
Teaching Hospital	14,840	21
SNF	5,873	371
K-12	4,739	51
Clinics and Community Hospitals	1,471	6
Employer	852	30

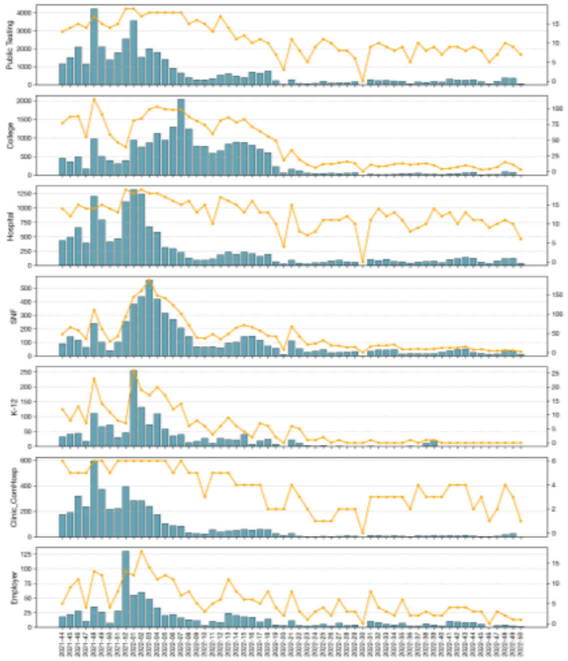

Supplemental Figure 6. Facility breakdown. Left is a table showing the different testing sectors included in this dataset. We show the total number of genomes within each sector along with the number of unique facilities. Right shows a weekly histogram of the number of genomes collected from each testing sector. The orange line shows the total number of unique facilities that were feeding the data collection during that week.

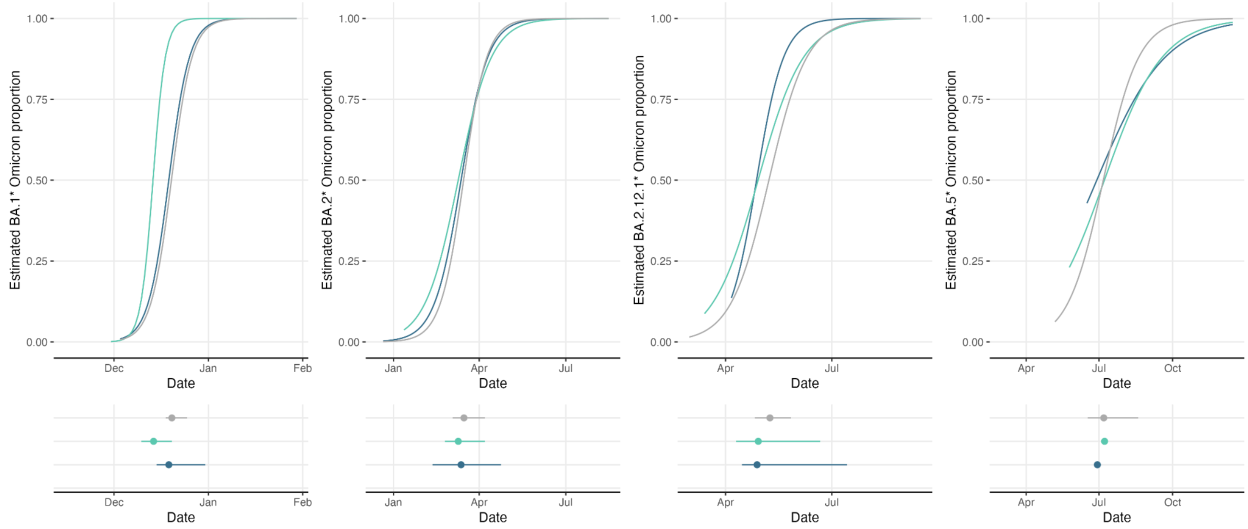

Supplemental Figure 7. We compared the growth trajectories of 4 lineages in 18-22 year olds in colleges and public testing to all of our public testing data. Plotted is the logistic regression curve. Below is the date at which we estimate each lineage to have reached 50% frequency with 95% CIs. For BA.5*, there was not enough data to fit 95% CIs on the 50% date.

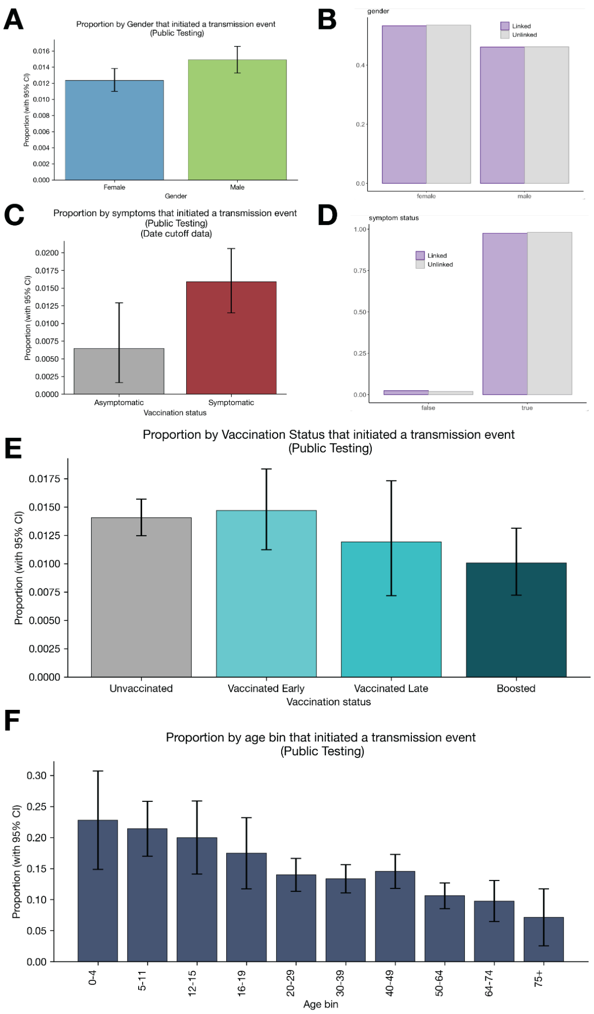

Supplemental Figure 8. Transmission by different features. A) Proportion of males vs females who initiated a transmission event. B) Proportion of males vs females who are in a network or not. C) Proportion of individuals who initiate a transmission event by presence of symptoms. We cut symptom data to only include those reported before January 1, 2022. We do not find a significant difference (p=0.1). D) Proportion of individuals with and without symptoms who are in a network or not. E) To better understand if individuals who were more recently vaccinated were less likely to initiate a transmission event, we split up the vaccinated cohort into two categories. Vaccinated early is those who were vaccinated between February 1, 2021 and

May 1, 2021 of vaccination being available. Vaccinated late is those that were vaccinated after booster doses became available (Nov 1, 2021). F) Proportion of individuals who initiated a transmission event by age bin.

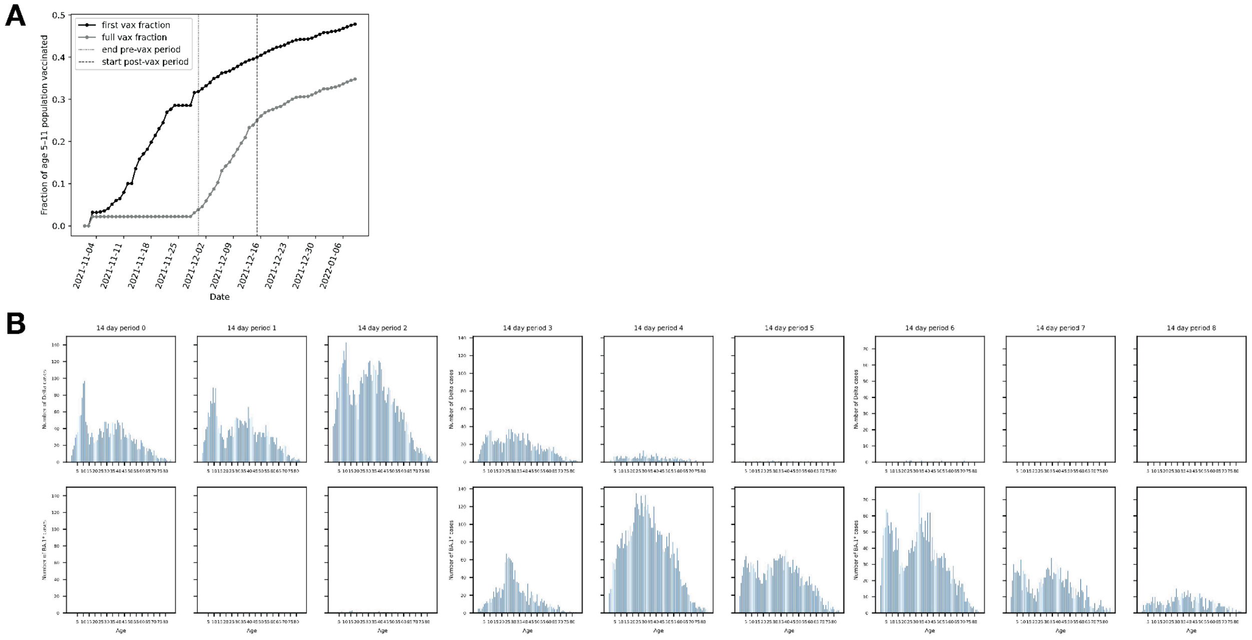

Supplemental Figure 9. A) Plotted is the percent of individuals aged 5-11 who received their first (black) and second (gray) vaccine doses after they became available. B) Two week histograms showing the percentage of each age in the Massachusetts population that was infected over time.

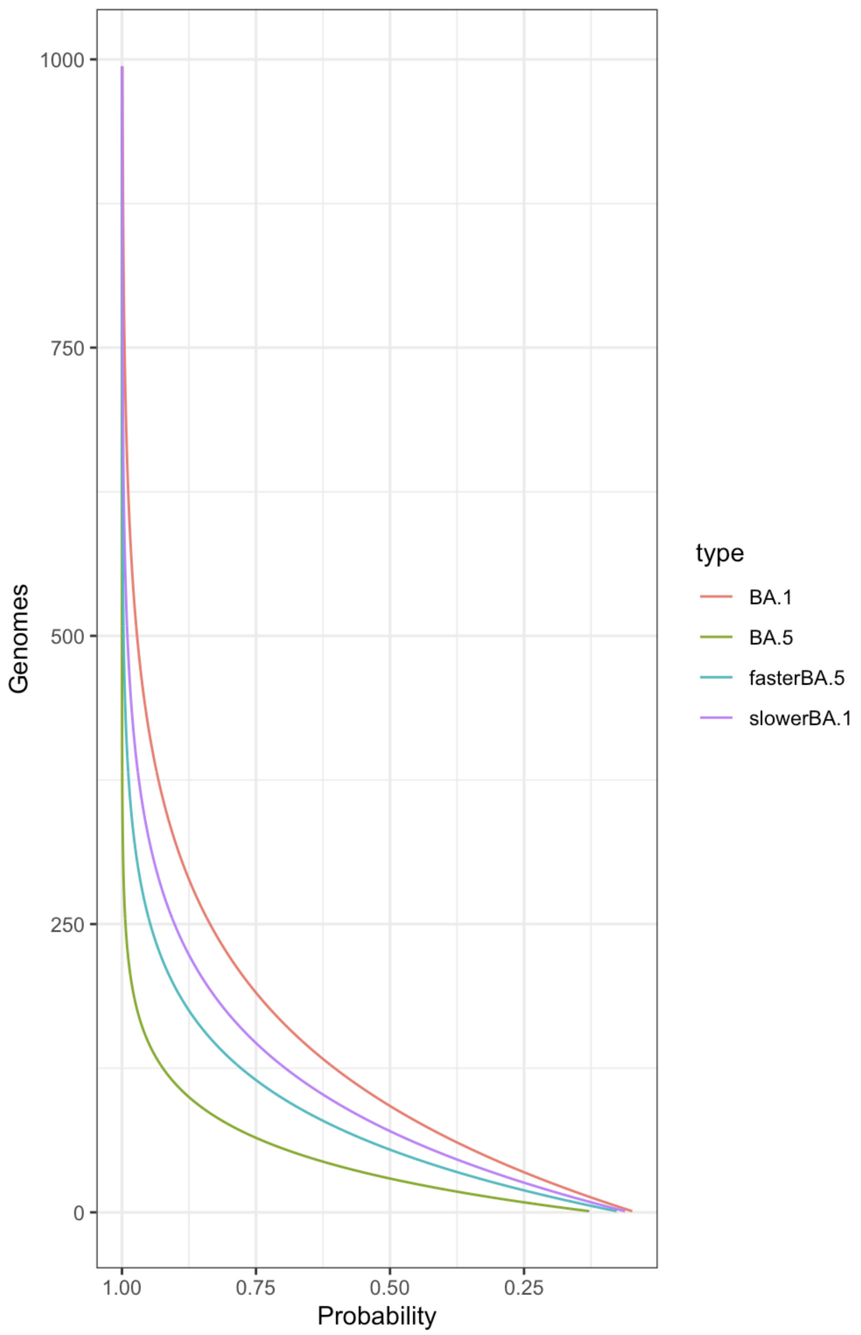

Supplemental Figure 10. Application of PhyloSamp on the number of high quality genomes needed to ensure initial detection before 1% prevalence of viruses with growth rates similar to those of BA.1 (a fast growing lineage in our dataset), BA.5 (a slow growing lineage in our dataset), and intermediate growth rates. The x-axis (plotted in reverse for comparability to Figure 4a) is the empirical probability of detecting the given lineage before 1% prevalence given that it logistically grows from 0.0001 prevalence. The y-axis is the number of high quality genomes generated per week.
